# Enhancement of standardized precipitation evapotranspiration index predictions by machine learning based on regression and soft computing for Iran’s arid and hyper–arid region

**DOI:** 10.1371/journal.pone.0319678

**Published:** 2025-03-18

**Authors:** Saeid Bour, Zahra Kayhomayoon, Farhad Hassani, Sami Ghordoyee Milan, Ommolbanin Bazrafshan, Ronny Berndtsson

**Affiliations:** 1 Department of Civil Engineering, Islamic Azad University Nour Branch, Nur, Iran; 2 Department of Geology, Payame Noor University, Tehran, Iran; 3 The University of Texas at Arlington, Arlington, Texas, United States of America; 4 Water Engineering Department, Faculty of Agricultural Technology, University College of Agriculture & Natural Resources, University of Tehran, Tehran, Iran; 5 Department of Natural Resources Engineering, Faculty of Agricultural Engineering and Natural Resources, University of Hormozgan, Bandar Abbas, Iran; 6 Centre for Advanced Middle Eastern Studies & Division of Water Resources Engineering, Lund University, Lund, Sweden; Euro-Mediterranean Center for Climate Change: Fondazione Centro Euro-Mediterraneo sui Cambiamenti Climatici, ITALY

## Abstract

Drought is a climate risk that affects access to safe water, crop development, ecological stability, and food production. Therefore, developing drought prediction methods can lead to better management of surface and groundwater resources. Similarly, machine learning can be used to find improved relationships between nonlinear variables in complex systems. Initially, the standardized precipitation evapotranspiration index (SPEI) was calculated, and then using large–scale signals such as large–scale climate signals (the North Atlantic Oscillation, the Arctic Oscillation, the Pacific Decadal Oscillation, and the Southern Oscillation Index), along with climatic variables including temperature, precipitation, and potential evapotranspiration, predictions were made for the period of 1966–2014. Several new machine learning models including Least Square Support Vector Regression (LSSVR), Group Method of Data Handling (GMDH), and Multivariate Adaptive Regression Splines (MARS) were used for prediction. The results showed that in estimating SPEI in moderately arid climates, the GMDH model with criteria (RMSE = 0.26, MAE = 0.17, NSE = 0.95 in validation) under scenario S1 (included all variables plus the SPEI of the previous month) performed better, while in arid and cold climates, the LSSVR model (RMSE = 0.22, MAE = 0.18, NSE = 0.95 in validation) under S1, and in arid and hot climate, the LSSVR model (RMSE = 0.29, MAE = 0.19, NSE = 0.93 in validation) under scenario S2 (included meteorological variables plus the SPEI of the previous month) had higher prediction accuracy. Although the MARS model was less accurate in validation, it showed higher accuracy during calibration compared to the other two models in all climates. The results showed that using large–scale signals for predicting SPEI was beneficial. It can be concluded that machine learning models are useful tools for predicting the SPEI drought index in different climates within similar ranges.

## 1. Introduction

Drought is caused by a temporary imbalance between the supply and demand of water, usually due to less rainfall than average [1]. Drought often develops slowly over time and has long–term effects [2]. Therefore, a challenge for managers and stakeholders is determining the onset of a drought period. Additionally, there are different severities of drought in terms of timing and spatial extent [3]. Drought is a natural phenomenon occurring in all climates [4]. Based on the operational definition, there are four main types of droughts. Meteorological drought indicates a lack of rainfall compared to the long–term average. Agricultural drought signifies moisture deficiency and reduced agricultural productivity. Hydrological drought denotes insufficient surface and groundwater supply. Another type is socio–economic drought where inadequate water supply leads to negative impacts on the environment, economy, and society [5]. Several indices have been developed by researchers to study drought, each addressing certain variables for the assessment. The efficiency of drought monitoring systems depends on the indices chosen according to the drought situation in the region. Over the years, numerous indices have been developed for monitoring drought in meteorological sectors [6].

The most important factor in drought situations is rainfall, but changes in temperature and consequent evaporation and transpiration can exacerbate or mitigate drought severity [7,8]. Various indices are based on rainfall or a combination of rainfall and evapotranspiration. Monitoring drought using different indices will undoubtedly yield different results. Therefore, since drought is a phenomenon dependent on multiple variables, it seems that, alongside precipitation, evapotranspiration should be considered, especially for arid and semi–arid regions [9]. Indices that consider both precipitation and evapotranspiration can be used to monitor current and future climatic changes. An appropriate index introduced for monitoring agricultural drought is the Standardized Precipitation Evapotranspiration Index (SPEI), which, due to considering the water balance, has a strong correlation with soil moisture and fast response to drought.

Previous studies have shown that SPEI is a robust indicator for drought monitoring and analysis [10–14]. Various models have been proposed for predicting drought indices. Models such as Autoregressive Integrated Moving Average (ARIMA), Markov chains, linear, and nonlinear regression models have been widely used by researchers [15–19]. Due to limitations such as the need for extensive data, heavy reliance on expert knowledge for identification, and sometimes complex approaches, machine learning methods have started to be developed in this area [20].

Machine learning models are capable of providing robust predictions in a time–efficient manner and with fewer variables [21]. These models are efficient in simulating environmental and hydrological phenomena [22–25]. In recent years, researchers have started to develop such models to predict various drought indices [26–29]. Machine learning models encompass a wide spectrum of methods, each with different performance based on the algorithms defined in their structure. However, one of the primary challenges we face in predicting drought is the use of local and regional data, such as rainfall observations and climatology. On the other hand, the limited length of historical data including uncertainties and errors necessitates researchers to continuously restructure prediction methods [7]. Over the past two decades, the use of climatic signals and remote sensing data have overcome some of these problems [7]. Climatic signals, with long recurrence periods, are sufficiently reliable and are used worldwide for predicting droughts, floods, minimum or maximum river flows, the onset of warm or cold seasons, etc. [30].

Several studies in recent years have shown that the trend of drought and salinity is related to large–scale climatic phenomena. These phenomena are defined as standardized numerical indices such as SOI, NAO, PDO, and NOI, which have been examined by researchers for their correlation and relationships with local hydroclimatic variables in different regions of the world. These indices are obtained through measurements of temperature and air pressure at various points in the atmosphere [31]. Several studies have utilized meteorological parameters and large–scale variables to study drought. For example, Deo et al. (2018) [32] predicted agricultural drought using climatic variables and signals and the Support Vector Regression (SVR) model in Australia. The results showed that the model had good capability in estimating drought, and there was no significant difference between observed and estimated drought characteristics. Tian et al. (2018) [33] address the prediction of agricultural drought using SPEI at different time scales and the indices niño southern oscillation, Western Pacific Subtropical High (WPHS(, and SVR. The results showed that SPEI–6 reflected soil moisture better than other variables, and WHPS mainly controlled the region’s temperature, with the addition of this variable improving the model’s accuracy. Morid et al. (2007) [34] predicted drought using an artificial neural network, precipitation, and the SOI and NAO indices in Tehran province. The results showed that precipitation was the best predictor, and the two selected signals had little effect on prediction. Additionally, the results of the correlation between the standardized precipitation index (SPI) and ENSO and PDO indices showed a strong relationship between SPI and the two mentioned climate signals at the time of drought occurrence [35].

Among various machine learning models, Multivariate Adaptive Regression Splines (MARS) and least square support vector regression (LSSVR) models are some of the most recent ones that have been utilized in various research studies. MARS can be used to provide regression relationships. The model has been successfully used in predicting groundwater response by Fernandes et al. (2024) [36], runoff prediction by Singh et al. (2022) [37], surface water quality index prediction by Trach et al. (2022) [38], and evaluation of the scour propagation rates around pipelines by Najafzadeh et al. (2022) [39]. LSSVR has also been evaluated in various time series studies such as the prediction of longitudinal dispersion coefficient of rivers by Arya Azar et al. (2023) [23], prediction of groundwater level by Kayhomayoon et al. (2023) [40], and evaluation by Kumar et al. (2023) in predicting river flow [41]. Along with these two models, Group Method of Data Handling (GMDH) is also based on deep learning. One of the capabilities of this model is to convert a complex data structures into simpler ones, which can be effective in estimating parameters such as SPEI. However, this aspect has so far received less attention in research. However, Table 1 presents a summary on studies for SPEI prediction using machine learning. Due to global warming, an increasing number of studies is focusing on drought prediction. Many of these studies have used meteorological variables in the estimation of the SPEI, e.g., Poornima and Pushpalatha (2019) [42], Dikshit et al. (2021) [43], and Mokhtar et al. (2021) [44]. Fewer studies have used data from satellite imagery to predict SPEI (e.g., Deo et al., 2018 [32]). To summarize, less studies have been made on SPEI prediction using climate variables and global climate indices. Thus, one of the novelties of this study is the utilization of the three machine learning methods; MARS, LSSVR, and GMDH for this purpose. Each of these methods has a unique structure and performance. Use of the MARS model is novel and the extraction of its regression relationship could prove beneficial in prediction of SPEI. However, questions persist regarding its accuracy compared to other machine learning methods such as LSSVR and GMDH, as well as the complexity of the extracted relationship. Therefore, the present study aimed to: (1) extract the most effective variables in predicting agricultural drought using MARS, LSSVR, and GMDH, and (2) compare the characteristics of observed and predicted droughts and introduce the best model for predicting agricultural drought in three climates; hyper–arid–moderate, arid–cold, and arid–hot climate in Iran. Hence, the novelty of this research compared to similar studies lies in considering the use of new machine learning models for predicting the SPEI index in these three typical climate areas, taking into account climate variables and remote sensing data.

**Table 1:** Summary of studies to predict SPEI using machine learning (2016–2024) compared to the present study.

Authors (year)	Region	Machine learning	Input variables
Maca and Pech (2016) [14]	USA	Artificial neural networks	SPEI with delays
Shang et al. (2016) [45]	China	Support vector machine Autoregressive integrated moving average	Precipitation Maximum temperature Minimum temperature Mean temperature wind speed Sunshine hours
Tian et al. (2018) [33]	China	Support vector machine	Precipitation Maximum temperature Minimum temperature Wind speed Mean relative humidity Daylight hours
Soh et al. (2018) [46]	Malaysia	Wavelet– autoregressive integrated moving average – artificial neural networks Wavelet–adaptive neuro–fuzzy inference system	SPEI with delays
Deo et al. (2018) [32]	Australia	Support vector machine	Precipitation Maximum temperature Minimum temperature Mean temperature Evapotransopiration Supplemented by climate indices (southern oscillation index, Pacific decadal oscillation, southern annular mode, and Indian Ocean dipole), and sea surface temperatures (Niño 3.0, 3.4, and 4.0)
Poornima and Pushpalatha (2019) [42]	India	Autoregressive integrated moving average Long short–term memory Artificial neural networks Support vector machine Wavelet analysis neural network	Maximum temperature Minimum temperature Maximum relative humidity Minimum relative humidity Precipitation Wind speed Sunshine Evapotranspiration
Abbasi et al. (2019) [47]	Iran	Gene expression programming	SPEI with delays
Fung et al. (2019) [27]	Malaysia	Wavelet–boosting– support vector machine Multi–input wavelet–fuzzy– support vector machine Weighted wavelet–fuzzy– support vector machine	SPEI with delays
Shamshirband et al. (2020) [29]	Iran	Support vector machine Gene expression programming M5 model trees	SPEI with delays
Khan et al. (2020) [28]	Pakistan	Support vector machine Artificial neural networks K–nearest neighbour	Mean temperature Precipitation SPEI with delays
Fung et al. (2020) [5]	Malaysia	Support vector machine Fuzzy– support vector machine Boosted–support vector regression	SPEI with delay of 1 month
Dikshit et al. (2021) [43]	Australia	Long short–term memory Random forests Artificial neural networks	Precipitation Maximum temperature Minimum temperature Mean temperature Vapour pressure Cloud cover Evapotranspiration
Mokhtar et al. (2021) [44]	China	Random forest Extreme gradient boost Convolutional neural network Long short–term memory	Precipitation Maximum temperature Minimum temperature Mean temperature Solar radiation Sunshine hours Wind speed at 2 m Relative humidity
Xu et al. (2021) [48]	China	Artificial neural networks Long short–term memory Support vector regression Least square– support vector regression Autoregressive integrated moving average – support vector machine Autoregressive integrated moving average – Long short–term memory	SPEI with delays
Ghasemi et al. (2021) [49]	Iran	Multi–layer perceptron neural network General regression neural network Gaussian process regression	Precipitation Mean temperature Relative humidity Aridity index
Tian et al. (2021) [50]	China	Random forest Long short–term memory Wavelet neural network Support vector machine	SPEI with delays
Karbasi et al. (2021) [51]	Iran	Gaussian process regression Cascade neural network Multi–layer perceptron neural network	Precipitation Maximum temperature Minimum temperature Wind speed Mean relative humidity Daylight hours
Lotifard et al. (2022) [52]	Iran	Random forest	SPEI with delays
Danandeh Mehr et al. (2023) [53]	Turkey	Convolutional long short–term memory Short–term meteorological drought forecasting	SPEI with delays
Present study	Iran	Multivariate adaptive regression splines Least square– support vector regression Group method of data handling	Precipitation Maximum temperature Minimum temperature Mean temperature Evapotranspiration Arctic oscillation North Atlantic oscillation Pacific Decadal Oscillation Southern oscillation index

## 2. Materials and methods

### 2.1. Study area

Iran is generally located in the arid and semi–arid region, however, with a wide variety of this general climate. Commonly, the north of Iran has highlands and mildly arid or humid areas, while the south, with the lowest elevation, is mainly characterized by moderate to severe aridity. This makes Iran constantly at risk of droughts and vulnerable to the effects of climate change. Among the different regions with different climatic characteristics, one study area was randomly selected for evaluation in each of these climatic region. Accordingly, climate analyses were performed using the modified extended de–Martone method [54] for 13 synoptic climatic stations with 48–year records (577 months). Table 2 shows the characteristics of the climatic stations in each of these selected regions. The three selected arid climates were: arid-cold, arid–hot, and hyper–arid–moderate. The areas can be said to be representative for each type of climate. As can be seen, 13 stations represent these climates. Six stations were located in arid-cold climate, three in arid-hot, and four in hyper–arid–moderate climate. In the arid–cold, arid–hot and hyper–arid–moderate climates, the mean annual precipitation varied between 161 and 319 mm, 22.5 and 248 mm, and 56 and 80 mm), respectively.

**Table 2:** Climatic stations and variables in arid and hyper–arid regions of Iran.

Station name	Elevation (m)	Mean annual precipitation (mm)	Mean annual temperature (°C)	Minimum mean monthly temperature (°C)	Climate type
Tabriz	1261	274	18.02	–3.74	Arid-cold
Torbat–E–Heydarieh	1451	264	19.52	–2.12	
Sabzevar	972	193	23.77	1.97	
Shahroud	1349	161	20.17	0.65	
Shiraz	1484	319	25.29	1.63	
Mashhad	999	253	20.92	–0.96	
Ahvaz	231	22.5	32.57	6.42	Arid–hot
Boushehr	9.00	248	30.13	8.20	
Dezful	260	143	29.14	6.20	
Bam	1070	58	27.5	3.25	Hyper–arid–moderate
Zabol	489	56	28.74	4.12	
Zahedan	1370	80	26.19	4.65	
Tabas	711	72	28.68	4.09	

**Fig 1:**
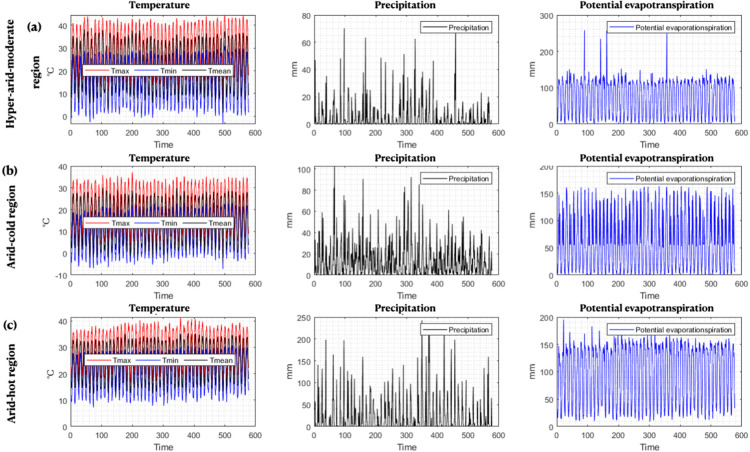
Time series of meteorological variables as input to models a) hyper–arid–moderate, b) arid–cold, c) arid–hot.

To predict the SPEI index, two data groups including meteorological data such as maximum temperature (T_max_), minimum temperature (T_min_), mean temperature (T_mean_), precipitation (Pr), and potential evapotranspiration (ETPc) and large–scale climatic signals (AO, NAO, PDO and SOI) were used at a monthly time step during the time period 1966–2014. The meteorological time series used in this study are presented in Fig 1. According to the figure, the highest T_max_ for arid–hot, hyper–arid–moderate and arid–cold climates was 41.4, 44.5 and 37.1°C, respectively. On average, T_max_ in these climates fluctuated in the range of 29.7, 29.8, and 20.7°C. T_min_ fluctuated between 7.3 and 30.9°C, –3.4 and 30.8°C, and –7.1 and 23.1°C for the mentioned climates, respectively. The highest mean annual precipitation for these climates was 242.6, 72.2, and 102.7 mm, respectively, which was less than the average annual precipitation for the entire Iran, equivalent to 250 mm. The largest annual potential evapotranspiration occurred in hyper–arid–moderate climate (256.6 mm), which averaged 73.8 mm per year in this climate. Among the three climates, the arid–hot climate had the highest average annual potential evapotranspiration (97.0 mm) (Fig 1).

**Fig 2:**
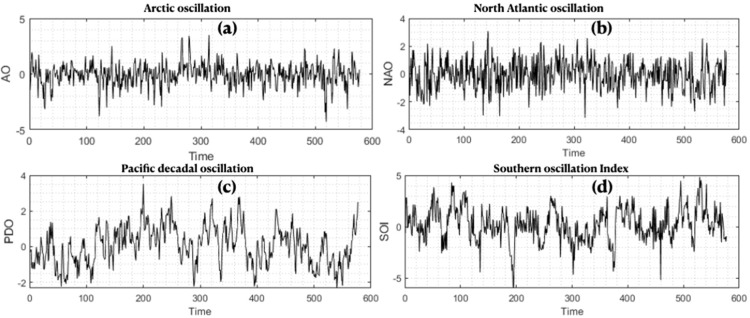
Time series of large–scale climate indices used in the modeling.

The meteorological variables were used together with large–scale climate indices in an attempt to improve SPEI prediction [55]. Fig 2 shows the corresponding large-scale climate indices that were used as input to the modeling after standardization and normalization. These were obtained from the NOAA website. The ENSO index is related to two indices, the Southern oscillation index (SOI), sea level pressure (SLP), and sea surface temperature (SST) in the equatorial Pacific ocean, where its negative and positive values indicate the cold and warm phases of ENSO or La Niña and El Niño conditions [56]. The Pacific decadal oscillation (PDO) is a climate oscillation pattern with its center of variability over the Pacific ocean and North america, and its measurements of SST and SLP are taken in the North pacific ocean (20° N) and in the northern United States [57]. The Arctic oscillation (AO) is the northern Hemisphere annular mode and is characterized by winds circulating the Arctic at a latitude of around 55^o^ N. The North Atlantic oscillation (NAO) consists of a north–south dipole of anomalies, with one center located over Greenland and the other center of opposite sign spanning the central latitudes of the North Atlantic between 35^o^ N and 40^o^N.

### 2.2. SPEI index

Developed by Vicente–Serrano et al. (2010) [58], the standardized precipitation evapotranspiration index (SPEI) is a widely used metric for evaluating drought conditions. This index takes into account precipitation, temperature, and potential evapotranspiration (PET). The SPEI combines the sensitivity of the Palmer drought severity Index (PDSI) to changes in evapotranspiration with the straightforward calculations and multiscale characteristics of the standardized precipitation index (SPI). As a result, it incorporates the attributes of both the SPI and the PDSI. To obtain the value of SPEI, the first step includes estimating the evapotranspiration for each month. Then, utilizing a straightforward water balance model, the difference between precipitation (P) and potential evapotranspiration (PET) for month *i* is calculated using:


$$D_{i}=p_{i}-PET_{i}$$ (1)

Calculating the SPEI is similar to the method used for computing the SPI, as it involves estimating cumulative probability values for the *D*_*i*_ values through “*i*” by fitting a probability density function. Due to the tendency of Di values to converge to negative values, two–parameter probability functions are unsuitable for this calculation. Vicente–Serrano et al. (2010) [58] found that the three–parameter log–logistic probability density function provided the best fit for the *D*_*i*_ values among the various functions tested. The general form of this probability density function is:


$$f(x)=\frac{\beta }{\alpha }{\left(\frac{n-\gamma }{\alpha }\right)}^{\beta -1}\left[1+{\left(\frac{n-\gamma }{\alpha }\right)}^{2}\right]$$ (2)

where α, β, and γ are the scale, shape, and main parameters, respectively, for the *D*_*i*_ values within the domain of ∞  <  *D*>  γ. The cumulative probability function of this distribution is:


$$F(x)={\left[1+{\left(\frac{\alpha }{X-\gamma }\right)}^{\beta }\right]}^{-1}$$ (3)

To estimate SPEI, we utilized the Classic Abramovich–Vastigan function according to:


$$SEPI=W-\frac{C_{0}+C_{1}W+C_{2}W^{2}}{1+d_{1}W+d_{2}W^{2}+d_{3}W^{3}}$$ (4)

where *W* is calculated using:


$$\begin{array}{l} \begin{array}{cc} for & p \end{array}\leq 0.5\\ W=\sqrt{-2\ln(p)} \end{array}$$ (5)

where *p* represents the probability of the specified values of *D* being exceeded. The values of *C*_*0*_, *C*_*1*_, and *C*_*2*_, as well as *d*_*1*_, *d*_*2*_, and *d*_*3*_, are constants. The SPEI is a standardized variable and can therefore be compared with other SPEI values in space and time. A SPEI value equal to zero corresponds to cumulative probabilities (*D*) of 0.05.

### 2.3. Machine learning models

Three machine learning models were utilized to estimate the SPEI–12 index, each of which is explained below. The models employed in this study included MARS, LSSVR, and GMDH, all of which possess unique structures and performance levels. Each of these methods offers specific features: MARS provides a robust regression relationship that is conducive to accurate predictions, LSSVR is an enhanced version of SVR that, despite its simplicity, yields highly accurate results in forecasting, and GMDH is a deep learning model that simplifies a complex structure through a series of steps.

#### 2.3.1. Least square support vector regression.

Introduced in 1999 [30], LSSVR aims to overcome some of the shortcomings of SVR since it provides us with lower computational complexity, higher performance, and lower runtime. Assume that {*x*_*k*_, *y*_*k*_}_*k* = 1_^*N*^ is training data where input and output data include *x*_*k*_ ∈  R^*N*^ and *y*_*k*_ ∈  R, respectively. The nonlinear regression function in the initial weighting space is:


$$y(x)=W^{T}\varphi (x)+b$$ (6)

where *W* and *b* represent the weight and bias of the regression function, respectively. *T* denotes the transpose symbol, and *φ*(*x*) is the nonlinear mapping of inputs in the high–dimensional feature space. In LSSVR, we are looking for a solution for the optimizing problem according to:


$$\min\;j(w,e)=\frac{1}{2}W^{2}W+\frac{1}{2}\gamma \sum_{k=1}^{N}e_{k}^{2}$$ (7)

where *e* indicates an error function and *γ* is a regulation parameter and controls the approximation function. The solution to Eqn. (7) is:


$$L(w,\;b,e,\alpha )j(w,e)-\sum_{i=1}^{N}\alpha_{i}\left\{W^{T}\varphi (\chi )+b+e_{k}-y_{k}\right\}$$ (8)

where *α* is the Lagrangian coefficient. The LSSVR model can be written in the form:


$$y(\chi )=\sum_{k=1}^{N}\alpha_{k}K(\chi ,\;\chi_{k})+b$$ (9)

where *K*(*χ*, χ_k_) is called the kernel function.

#### 2.3.2. Multivariate adaptive regression splines (MARS).

**Fig 3:**
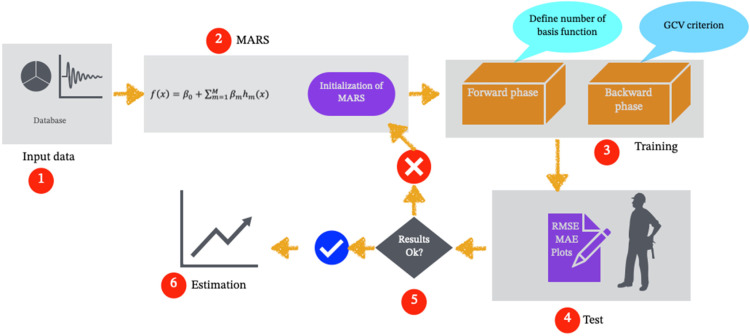
Schematic of MARS model.


Presented by Friedman (1991) [59], MARS is a non–parametric regression method for flexible regression modeling of high–dimensional data. The general form of this model can be expressed as [60]:


$$f\left(x\right)=\beta_{0}+\overset{M}{\underset{m=1}{\sum }}\beta_{m}h_{m}\left(x\right)$$ (10)

where *x* is a variable and there is a constant value in this model called a node. For each variable, the identified node will differ, and the coefficient *β*_*m*_ varies with each state of *h*_*m*_. The number and location of the nodes are determined through a forward–backward step–by–step process (Fig 3). During the forward phase, many nodes are generated, while in the backward phase, the nodes that contribute less to the overall fit are removed. At each step, from all the lines of each basis function, a linear process is selected that minimizes the defects of the fitted model. Basic functions that yield the minimum possible values to the model undergo elimination steps, and the optimal model is then selected. The loss of fit index used is based on a standardized criterion:

$$GCV\left(M\right)=\frac{1}{n}\overset{n}{\underset{i=1}{\sum }}{\left(y_{i}-\hat{y}\right)}^{2}/{\left(1-\frac{C\left(M\right)}{n}\right)}^{2}$$ (11)

where $\hat{y}$ is the dependent value predicted by the model, *n* is the number of observations in the data series, *M* is the number of non–constant terms in the model, and *C*(*M*) is the error function. The purpose of *C*(*M*) is to compensate for the complexity of the model; in this situation, excessive fitting is avoided to increase the efficiency of the model.

#### 2.3.3. Group method of data handling (GMDH).

**Fig 4:**
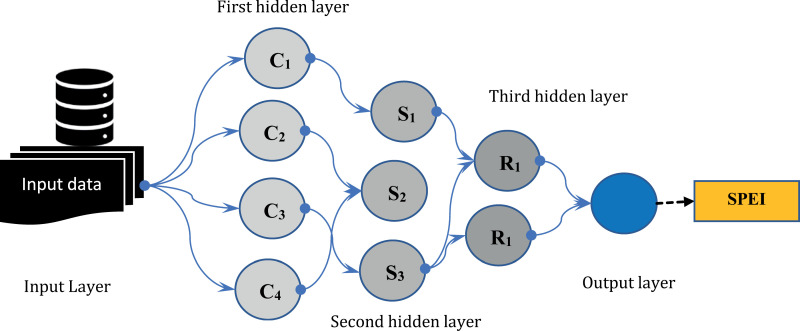
Schematic of GMDH model.


GMDH is a model designed to address complex and nonlinear problems [61]. It constructs a self–organizing model capable of handling prediction, classification, and other system issues. The GMDH method automatically determines the number of neurons, hidden layers, effective input variables, and the network structure [62] (Fig 4). This method assumes that the relationship between the system’s input and output can be represented by a high–order equation:

$$y=a_{0}+\overset{m}{\underset{i=1}{\sum }}a_{i}x_{i}+\overset{m}{\underset{i=1}{\sum }}\overset{m}{\underset{j=1}{\sum }}a_{ij}x_{i}x_{j}+\overset{m}{\underset{i=1}{\sum }}\overset{m}{\underset{j=1}{\sum }}\overset{m}{\underset{k=1}{\sum }}a_{ijk}x_{i}x_{j}x_{k}+\ldots$$ (12)

where X (x_1_, x_2_, …, x_m_) is the input vector and a (a_1_, a_2_, …, a_m_) is the vector of coefficients or weights and y is the output. GMDH includes four steps. The first step includes generating new variables z_1_, z_2_,..., z_n_. For all input variables (x_1_, x_2_, …, x_m_), second–order regression equations are defined using the training data according to:


$$z=c_{1}+c_{2}x_{i}+c_{3}x_{f}+c_{4}x_{i}^{2}+c_{5}x_{f}^{2}+c_{6}x_{i}x_{f}$$ (13)

Then, the error of the neurons is calculated to distinguish active and inactive neurons. This is followed by removing inactive neurons. In the third step, new variables are generated as the input of the regression equations. The second step is then repeated and the outputs that have the best approximation to the desired output are selected. The GMDH is finally assessed using the validation data (Fig 4).

### 2.4. Research methodology

**Fig 5:**
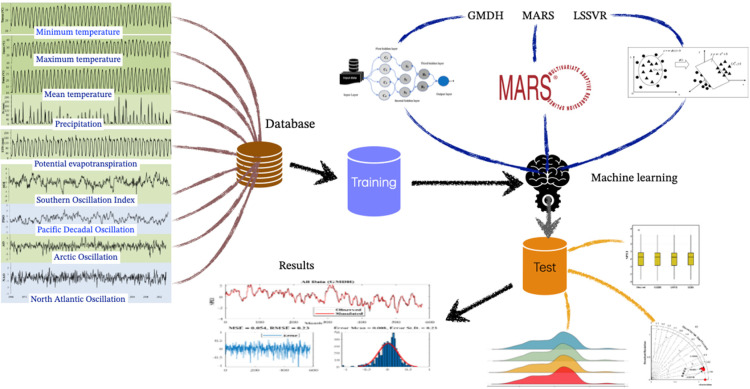
Schematic representation of the stages involved in predicting SPEI–12.

The research methodology is presented in Fig 5. To predict the SPEI–12 index, first, the parameters affecting it, which vary over time, need to be selected. Therefore, meteorological variables including maximum temperature (T_max_), minimum temperature (T_min_), mean temperature (T_mean_), precipitation (Pr), and potential evapotranspiration (ETPc), as well as large–scale climate indices including AO, NAO, PDO, and SOI for the three aforementioned climates during 1966–2014, were chosen. Since different combinations of these variables as inputs to the model yield different results, various patterns of variable combinations were formulated. Three different scenarios were considered. The scenarios were arranged based on a combination of meteorological variables and large–scale climate indices to evaluate the accuracy of predictions using various parameters. The scenarios were created using two categories of meteorological data and large–scale signal data (Table 3). According to the table, the first scenario (S1) included all variables plus the SPEI of the previous month, the second scenario (S2) included meteorological variables plus the SPEI of the previous month, and the third scenario (S3) included large–scale indices plus the SPEI of the previous month. It should be noted that the data were considered on a monthly basis, with a total of 577 data points, of which 70% were used for model calibration and 30% for validating the models. Furthermore, the SPEI–12 index in three climates was estimated using the aforementioned scenarios and machine learning models (LSSVR, GMDH, and MARS) to select the most suitable model and the variables that have the greatest impact on SPEI (Fig 5).

**Table 3:** Development of input scenarios for SPEI estimation.

	Input										Output
	Meteorological variables					Large–scale climate indices					
Pattern	T_max_	T_min_	T_mean_	Pr	ETP_c_	AO	NAO	PDO	SOI	SPEI_n–1_	SPEI
S1	✓	✓	✓	✓	✓	✓	✓	✓	✓	✓	
S2	✓	✓	✓	✓	✓	×	×	×	×	✓	
S3	×	×	×	×	×	✓	✓	✓	✓	✓	

#### 2.3.4. Error evaluation criteria for machine learning models.

The accuracy of the models and scenarios developed was assessed using error evaluation criteria. Root mean square error (RMSE) (Eqn. 14), mean absolute error (MAE) (Eqn. 15), and Nash–Sutcliffe efficiency (NSE) (Eqn. 16) we re employed to assess the performance of machine learning methods across various scenarios [50] (Tian, 2021):


$$\text{RMSE}=\sqrt{\frac{\sum_{i=1}^{n}{\left(x_{i}^{obs}-x_{i}^{sim}\right)}^{2}}{n}}0\leq RMSE\leq \infty$$ (14)

$$\text{NSE}=1-\frac{\sum_{i=1}^{n}{\left(x_{i}^{obs}-x_{i}^{sim}\right)}^{2}}{\sum_{i=1}^{n}{\left(x_{i}^{obs}-\bar{x^{obs}}\right)}^{2}}-1\leq NSE\leq 1$$ (15)

$$\text{MAE}=\frac{\sum_{i=1}^{n}\left|x_{i}^{obs}-x_{i}^{sm}\right|}{n}0\leq MAE\leq \infty$$ (16)

where *x* ^*obs*^ is the observed value, *x*^*sim*^ is the simulated value, $\bar{x^{obs}}$ is average of the observed value, and n is the number of samples. The closer the RMSE and MAE values are to zero, the better the performance of the models. On the other hand, values close to one for NSE indicate good performance.

We used Eqn. (17) and (18) to analyze the uncertainty in the output results from the models and calculating uncertainty intervals. The intervals show upper and lower bounds for the estimated SPEI values related to acceptable uncertainty ranges. In this case, *σ* represents the standard deviation and *µ * represents the mean output over 100 model runs.


$$\text{Upper band }\left(95\text{\%}\right)=(\text{mean of prediction}+\left(1.96\times \text{standard deviation of prediction }\right)$$ (17)

$$\text{Lower band }\left(95\text{\%}\right)=(\text{mean of prediction}–\left(1.96\times \text{standard deviation of prediction }\right)$$ (18)

## 3. Results

**Fig 6:**
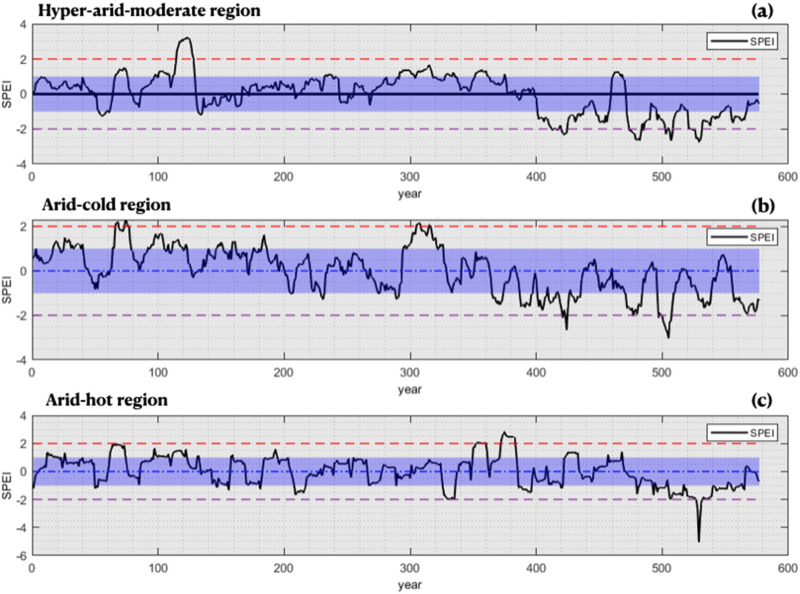
SPEI–12 index for the three studied climates a) hyper–arid–moderate region, b) arid–cold region, c) arid–hot region.

Descriptive statistics of all input variables are provided in Table 4. In Fig 6, the SPEI–12 index is presented for each climate. As shown in Fig 6a, from 1966 to 2000, the index indicates that the hyper–arid–moderate climate was in a wet period and had almost entered into a drought since 2000. In the arid–cold climate, as depicted in Fig 6b, a drought period started around 1998, with the most severe drought experienced in 2006 and 2009. According to Fig 6c, the arid–hot climate experienced less drought compared to the hyper–arid–moderate and arid–cold climates, and the SPEI–12 index followed more of a sinusoidal pattern. Based on the graph, the drought period for the climate in question started around 2008 and experienced its most severe drought in 2010. These changes have been due to a decrease in precipitation and a relative increase in temperature since around 1995.

**Table 4:** Descriptive statistics of model input variables used in the different climates.

	Variable	T_max_ (°C)	T_min_ (°C)	T_mean_ (°C)	Pr (mm)	ETP_c_ (mm)	AO	NAO	PDO	SOI
Hyper–arid–moderate	Max	44.5	30.8	38.2	72.2	256.57	3.495	3.06	3.51	4.8
	Min	8.10	–3.40	1.7	0.00	0.05	–4.266	–3.14	–2.33	–6.0
	St. D.	9.56	9.32	9.55	10.14	50.23	1.01	1.05	1.07	1.64
	Mean	29.76	14.76	22.54	4.66	73.81	–0.05	–0.03	0.06	0.21
	Skew	–0.24	–0.03	–0.14	3.36	0.01	–0.29	–0.09	0.11	–0.18
Arid–cold	Max	37.1	23.10	30.1	102.7	163.25	3.495	3.06	3.51	4.8
	Min	0.80	–7.10	–3.9	0.00	0.01	–4.266	–3.14	–2.33	–6.0
	St. D.	9.97	8.370	9.25	16.49	51.37	1.01	1.05	1.07	1.64
	Mean	20.7	8.920	14.69	13.37	60.16	–0.05	–0.03	0.06	0.21
	Skew	–0.20	–0.02	–0.12	2.05	0.53	–0.29	–0.09	0.11	–0.18
Arid–hot	Max	41.4	30.9	35.2	242.6	195.21	3.495	3.06	3.51	4.8
	Min	15.8	7.30	11	0.00	9.35	–4.266	–3.14	–2.33	–6.0
	St. D.	7.04	6.71	6.85	40.61	54.05	1.01	1.05	1.07	1.64
	Mean	29.6	19.99	24.80	20.99	96.98	–0.05	–0.03	0.06	0.21
	Skew	–0.30	–0.10	–0.21	2.62	–0.32	–0.29	–0.09	0.11	–0.18

Before discussing the model results, it is important to present the optimal variable values for each model. In the LSSVR, the kernel function was considered Gaussian, with the optimal parameters σ^2^ and γ of 5.36 and 136.03, respectively. In the GMDH, the maximum number of neurons per layer was set to 20, the maximum number of layers to 5, and the selection pressure α to 0.6. In the MARS, the number of basic functions (NBF) varied for each scenario and climate condition, with the highest degree of interaction in the final model being 2. Scenarios S1 and S2 in the arid–cold climate and scenario S1 in the arid–hot climate required a higher number of functions to achieve the optimal solution (Table 5).

**Table 5:** Generalized cross validation values and the number of basic functions used in the MARS model.

Climate	Scenario	GCV	No. of functions
Hyper–arid–moderate	1	0.0465	9
	2	0.0478	17
	3	0.0566	11
Arid–cold	1	0.0544	23
	2	0.0543	26
	3	0.0856	8
Arid–hot	1	0.0682	23
	2	0.0699	19
	3	0.1517	14

The number of functions used in the MARS model for each climate is shown in Table 6. As can be seen, about 17 functions have been extracted for hyper–arid–moderate climates. For the other two climates, 23 functions were extracted. In the hyper–arid–moderate climate, the extracted functions were related to the S2 scenario, in which meteorological parameters and SPEI_n–1_ were involved. Also, in arid – cold climate, the extracted functions were obtained from the S1 scenario, in which the results were suitable than other scenarios. Finally, in the arid–hot climate, the S1 scenario, which included all parameters, had better estimation accuracy. According to extractive functions, it is observed that some functions require the implementation of the previous function. For example, the third function requires the implementation of the second function in a arid – hot climate. This relationship has also been observed for cold climates (*λ*_*3*_), demonstrating that some functions rely on each other. This relationship can be linked to fluctuations in precipitation and the SPEI value. For example, functions *λ*_*9*_, *λ*_*10*_, and *λ*_*14*_ are influenced by the outcomes of functions two and four. Therefore, cosidering into account SPEI (n–1) variables and precipitation can improve estimate accuracy.

**Table 6:** Number of functions used in MARS model for each climate.

Hyper–arid–moderate (17 functions)	Arid–cold (23 functions)	Arid–hot (23 functions)
*λ*_*1*_* = max (0,SPEI*_*n–1*_ *–1.1213)*	*λ*_*1*_ *= max(0, SPEI*_*n–1*_ +*0.40208)*	*λ*_*1*_ *= max(0, SPEI*_*n–1*_ *–1.4446)*
*λ*_*2*_ * = max(0, Pr –1.4) × max(0, 50.8 –ETPc)*	*λ*_*2*_ *= max(0, Pr –44.9)*	*λ*_*2*_ *= max(0, 1.4446 – SPEI*_*n–1*_)
*λ*_*3*_ *= max(0, Precipitation –1.4) × max(0, ETPc –47.1)*	*λ*_*3*_ *=* λ_*1*_ *× max(0, Precipitation –23.3)*	*λ*_*3*_ *=* λ_*2*_*× max(0, Pr –0.3)*
*λ*_*4*_ *= max(0, Pr –1.4) × max(0, 47.1 –ETPc)*	*λ*_*4*_ *= max(0, SPEI*_*n–1*_ +*0.3094)*	*λ*_*4*_ *= max(0, Pr –1.3)*
*λ*_*5*_ *= max(0, Pr –40)*	*λ*_*5*_ *= max(0, –0.402 – SPEI*_*n–1*_*) × max(0, T*_*max*_ *–9.9)*	*λ*_*5*_ *= max(0, 1.3 –Pr) × max(0, 26.4 – T*_*mean*_)
*λ*_*6*_ *= max(0, 1.1 –SPEI*_*n–1*_*) × max(0, 97.7 –ETPc)*	*λ*_*6*_ *= max(0, –0.402 – SPEI*_*n–1*_*) × max(0, 9.9 – T*_*max*_)	*λ*_*6*_ *= max(0, 30.1 –T*_*max*_)
*λ*_*7*_ *= max(0, Pr –1.4) × max(0, SPEI*_*n–1*_ *–0.34)*	*λ*_*7*_ *= max(0, –0.3094 – SPEI*_*n–1*_*) × max(0, T*_*max*_ *–8)*	*λ*_*7*_ *= max(0, 1.3 –Pr) × max(0, NAO –1.67)*
*λ*_*8*_ *= max(0, 40 –Pr) × max(0, Tmin –1.9)*	*λ*_*8*_ *= max(0, –0.3094 – SPEI*_*n–1*_*) × max(0, 8 – T*_*max*_)	*λ*_*8*_ *= max(0, 23.9 – T*_*mean*_)
*λ*_*9*_ *= max(0, 40 –Pr) × max(0, T*_*mean*_ *–25.3)*	*λ*_*9*_ *= max(0, 44.9 –Pr) × max(0, –0.40 – SPEI*_*n–1*_)	*λ*_*9*_ *=* λ_*2*_*× max(0, SOI +0.5*)
*λ*_*10*_ *= max(0, 40 –Pr) × max(0, 25.3 –T*_*mean*_)	*λ*_*10*_ *= max(0, 44.9 –Pr) × max(0, 25.7 –T*_*mean*_)	*λ*_*10*_ *=* λ_*2*_ *× max(0, –0.5 –SOI)*
*λ*_*11*_ *= max(0, 1.1213 –SPEI*_*n–1*_*) × max(0, T*_*max*_ *–22.8)*	*λ*_*11*_ *= max(0, –0.30 – SPEI*_*n–1*_*) × max(0, T*_*mean*_ *–2.2)*	*λ*_*11*_ *=* λ_*4*_ *× max(0, 9.2 – T*_*min*_)
*λ*_*12*_ *= max(0, 1.1213 –SPEI*_*n–1*_*) × max(0, T*_*mean*_ *–24)*	*λ*_*12*_ *= max(0, –0.31 – SPEI*_*n–1*_*) × max(0, 2.2 – T*_*mean*_)	*λ*_*12*_ *=* λ_*4*_ *× max(0, 15.5 – T*_*mean*_)
*λ*_*13*_ *= max(0, 1.1213 –SPEI*_*n–1*_*) × max(0, T*_*mean*_ *–17.7)*	*λ*_*13*_ *= max(0, –0.4 –SPEI*_*n–1*_*) × max(0, T*_*min*_ +*2.1)*	*λ*_*13*_ *= max(0, 1.3 –Pr) × max(0, AO –1.3)*
*λ*_*14*_ *= max(0, 15.5 –T*_*min*_*) × max(0, T*_*mean*_ *–24.8)*	*λ*_*14*_ *= max(0, –0.30 – SPEI*_*n–1*_*) × max(0, T*_*min*_ +*2.1)*	*λ*_*14*_ *= max(0, 1.076 –AO)*
*λ*_*15*_ *= max(0, 15.5 –T*_*min*_*) × max(0, T*_*max*_ *–31.5)*	*λ*_*15*_ *= max(0, 1.1 –SOI) × max(0, 2.1 –Pr)*	*λ*_*15*_ *=* λ_*6*_ *× max(0, –0.24 –AO)*
*λ*_*16*_ *= max(0, 15.5 –T*_*min*_*) × max(0, ETPc –57.465)*	*λ*_*16*_ *= max(0, 1.1 –SOI) × max(0, T*_*max*_ *–29.8)*	*λ*_*16*_ *= max(0, –1.05 –AO)*
*λ*_*17*_ *= max(0, 1.12 –SPEI*_*n–1*_*) × max(0, 9.1 –T*_*min*_)	*λ*_*17*_ *= max(0, 44.9 –Pr) × max(0, ETPc –4.51)*	*λ*_*17*_ *= max(0, AO* +*1.0) × max(0, Pr –54)*
	*λ*_*18*_ *= max(0, ETPc –5.1) × max(0, T*_*mean*_ *–26.8)*	*λ*_*18*_ *= max(0, AO* +*1.05) × max(0, 54 –Pr)*
	*λ*_*19*_ *= max(0, ETPc –5.18) × max(0, 26.8 –T*_*mean*_)	*λ*_*19*_ *=* λ_*14*_ *× max(0, NAO –1.54)*
	*λ*_*20*_ *= max(0, ETPc –44.832)*	*λ*_*20*_ *= max(0, 77.2 –Pr)*
	*λ*_*21*_ *= max(0, 44.832 –ETPc)*	*λ*_*21*_ *=* λ_*14*_ *× max(0, Pr –139.9)*
	*λ*_*22*_ *= max(0, T*_*mean*_ *–22)*	*λ*_*22*_ *= λ*_*14*_ *× max(0, –1.806 –AO)*
	*λ*_*23*_ *= max(0, 22 – T*_*mean*_)	*λ*_*23*_ *= max(0, x2 –10.4) × max(0, –0.8 –AO)*

Finally, by summing the functions of the above table in each climate, the final extracted relationship for each climate was obtained. As can be seen, all three climates have a separate relationship, where Eqn. (19) represents the hyper–arid–moderate climate. In this equation, the constant value wais 0.288. Similarly, Eqn. (20) and (21) represent the extraction equation for arid–cold and arid–hot climates, respectively. The high number of functions in both dry–hot and dry–cold climates is attributed to the presence of SPEI fluctuations relative to zero. In contrast, in the dry–cold climate with low fluctuations, fewer functions were utilized. In both mentioned equations, the constant value of the equation is positive and for arid–cold and arid–hot climates were 0.375 and 1.147, respectively.


$$SPEI_{\left(Hyper–arid–moderate\right)}=0.288+0.97\lambda_{1}+0.009\lambda_{2}+0.0006\lambda_{3}–0.009\lambda_{4}–0.028\lambda_{5}–0.014\lambda_{6}–0.008\lambda_{7}+0.0012\lambda_{8}–0.001\lambda_{9}+0.001\lambda_{10}–0.045\lambda_{11}+0.121\lambda_{12}–0.084\lambda_{13}+0.65\lambda_{14}–0.408\lambda_{15}+0.0016\lambda_{16}+0.041\lambda_{17}$$ (19)

$$SPEI_{\left(Arid–cold\right)}=0.375+3.85\lambda_{1}+0.0235\lambda_{2}–0.0072\lambda_{3}–2.91\lambda_{4}+0.601\lambda_{5}–0.818\lambda_{6}–0.987\lambda_{7}+1.27\lambda_{8}+0.0159\lambda_{9}–0.0005\lambda_{10}+0.786\lambda_{11}–0.651\lambda_{12}–0.778\lambda_{13}+0.3460\lambda_{14}+0.0267\lambda_{15}–0.0171\lambda_{16}–0.00013\lambda_{17}+0.003\lambda_{18}–0.0015\lambda_{19}+0.006\lambda_{20}–0.0321\lambda_{21}–0.1696\lambda_{22}+0.0372\lambda_{23}$$ (20)

$$SPEI_{\left(Arid–hot\right)}=1.1468+1.002\lambda_{1}–1.02\lambda_{2}+0.0021\lambda_{3}+0.0108\lambda_{4}–0.0452\lambda_{5}+0.1405\lambda_{6}–0.663\lambda_{7}–0.2165\lambda_{8}+0.0223\lambda_{9}+0.0394\lambda_{10}–0.0074\lambda_{11}+0.0013\lambda_{12}+0.937\lambda_{13}–0.380\lambda_{14}+0.064\lambda_{15}–0.658\lambda_{16}–0.00359\lambda_{17}–0.0060\lambda_{18}+0.682\lambda_{19}+0.0132\lambda_{20}–0.005\lambda_{21}+0.3637\lambda_{22}+0.0500\lambda_{23}$$ (21)

To evaluate the results of the models, evaluation metrics including RMSE, MAE, and NSE were utilized. These results for both calibration and validation are presented in Table 7. As mentioned, three different input scenarios were considered, which included ground station data, satellite data, and SPEI with a one–month lag (S1), ground station data, and SPEI with a one–month lag (S2), and satellite data and SPEI with a one–month lag (S3). Based on the results of the evaluation criteria, the proposed models have estimated the SPEI–12 index with high accuracy under different scenarios. For the hyper–arid–moderate climate, different scenarios have led to high accuracy of each model. In this climate, the results of the criteria for all three scenarios of the models are very close to each other.

**Table 7:** Qualitative statistics for the models used in the study. Selected scenarios are bolded and highlighted.

Climate	Models	Scenarios	Calibration			Validation		
RMSE	MAE	NSE	RMSE	MAE	NSE
Hyper–arid–moderate	LSSVR	S1	0.20	0.13	0.97	0.26	0.17	0.94
		S2	0.24	0.16	0.96	0.28	0.18	0.93
		S3	**0.25**	**0.16**	**0.95**	**0.24**	**0.17**	**0.96**
	**GMDH**	S1	**0.22**	**0.15**	**0.96**	**0.26**	**0.17**	**0.95**
		S2	0.24	0.16	0.96	0.26	0.18	0.94
		S3	0.25	0.17	0.95	0.27	0.19	0.95
	MARS	S1	0.20	0.13	0.95	0.27	0.19	0.96
		S2	**0.19**	**0.13**	**0.96**	**0.27**	**0.19**	**0.97**
		S3	0.23	0.16	0.94	0.30	0.21	0.96
Arid–cold	**LSSVR**	S1	**0.23**	**0.17**	**0.95**	**0.22**	**0.18**	**0.95**
		S2	0.22	0.17	0.96	0.24	0.18	0.94
		S3	0.29	0.22	0.92	0.26	0.18	0.94
	GMDH	S1	**0.24**	**0.18**	**0.95**	**0.22**	**0.16**	**0.96**
		S2	0.23	0.17	0.95	0.25	0.19	0.94
		S3	0.29	0.21	0.92	0.26	0.20	0.94
	MARS	S1	**0.20**	**0.15**	**0.95**	**0.33**	**0.25**	**0.93**
		S2	0.19	0.15	0.96	0.40	0.28	0.89
		S3	0.28	0.20	0.92	0.36	0.28	0.91
Arid–hot	**LSSVR**	S1	0.31	0.19	0.92	0.34	0.19	0.88
		S2	**0.29**	**0.17**	**0.92**	**0.29**	**0.19**	**0.93**
		S3	0.40	0.26	0.85	0.38	0.24	0.88
	GMDH	S1	0.29	0.19	0.93	0.34	0.23	0.89
		S2	**0.30**	**0.20**	**0.92**	**0.31**	**0.20**	**0.91**
		S3	0.40	0.25	0.86	0.40	0.24	0.86
	MARS	S1	0.22	0.14	0.95	0.41	0.22	0.83
		S2	**0.23**	**0.14**	**0.94**	**0.35**	**0.20**	**0.91**
		S3	0.35	0.24	0.88	0.61	0.34	0.74

The LSSVR model, by using satellite data with (RMSE = 0.24, MAE = 0.17, NSE = 0.96 for validation data(, has the most accuracy among the scenarios. This model, using scenario 1, has estimated the calibration data with criteria (RMSE = 0.20, MAE = 0.13, NSE = 0.97), which has the most accuracy compared to the other two scenarios. Also, in this scenario, the prediction accuracy of the validation data (RMSE = 0.26, MAE = 0.17, NSE = 0.94) is also very close to the selected scenario. In this climate, GMDH model using all parameters of (AO, NAO, PDO, SOI) and ground station (Tmax, Tmin, Tmean, Pr, ETPc) with criteria (RMSE = 0.22, MAE = 0.15, NSE = 0.96 for calibration data and RMSE = 0.26, MAE = 0.17, NSE = 0.95) for validation data) has been more accurate in predicting SPEI than the other two models. The MARS model has estimated calibration data more accurately than the other two models, so that the most accuracy of this model is obtained among calibration data in the second scenario with the criteria (RMSE = 0.26, MAE = 0.17, NSE = 0.95). The mentioned model has almost the same accuracy in predicting the validation data in scenario 1 and 2, but the highest accuracy of the model has been achieved in scenario 2 (RMSE = 0.27, MAE = 0.19, NSE = 0.97).

In the arid and cold climate, the highest accuracy of the models was obtained by utilizing S1 scenario data. In this climate, the LSSVR model showed higher accuracy compared to the two GMDH and MARS models with criteria (RMSE = 0.23, MAE = 0.17, NSE = 0.95 for calibration data) and (RMSE = 0.22, MAE = 0.18, NSE = 0.95 for validation data). Additionally, the GMDH model had a very close performance to the LSSVR model in predicting validation data (RMSE = 0.22, MAE = 0.16, NSE = 0.96), but its performance decreased in predicting calibration data. Although the MARS model estimated the calibration data with an accuracy of (RMSE = 0.20, MAE = 0.15, NSE = 0.95) in the selected scenario, it predicted the validation data with a relatively higher error in all three scenarios (Table 7). In the arid and hot climate, using variables from scenario S2 led to the highest accuracy of the models in estimating SPEI. In predicting the calibration data for all three scenarios, the models showed very close performance. In these data, the models had better performance using scenarios S1 and S2 compared to scenario S3, with the RMSE range in scenarios S1 and S2 being between 0.22 and 0.31, and in scenario S3, between 0.35 and 0.4. It should be noted that in estimating the calibration data, the MARS model had the highest accuracy using scenario S1 (RMSE = 0.22, MAE = 0.14, NSE = 0.95). Overall, in this climate, the LSSVR model provided a better estimation of SPEI–12 compared to the other two models. A general comparison of the models in the three studied climates shows that the MARS model had the highest accuracy in estimating SPEI–12 in the arid and cold climate under scenario S2, and for validation data, the LSSVR model in the arid and cold climate under scenario S1 exhibited higher accuracy. Consequently, it can be stated that large–scale climatic signals had a positive impact on increasing the accuracy of predictive models (Table 7).

**Fig 7:**
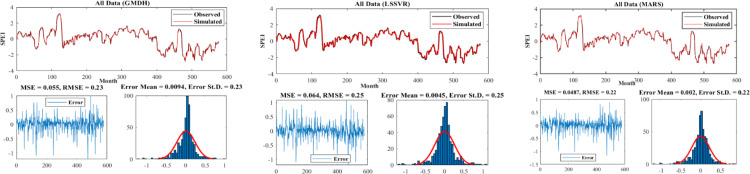
Time series plot and model errors for the hyper–arid–moderate climate.

**Fig 8:**
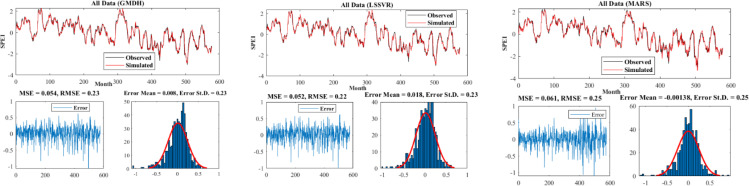
Time series plot and model errors for the arid–cold climate.

**Fig 9:**
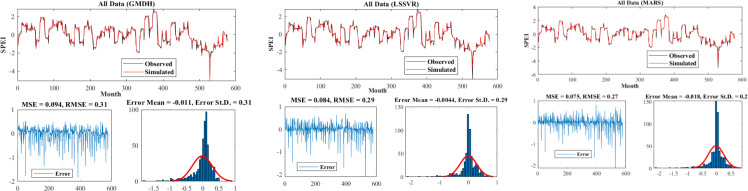
Time series plot and model errors for the arid–hot climate.

Figs 7–9 present the time series plot of observational and simulated data for the selected scenarios, error plots, and the distribution of data for each model in three different climates. As evident from the time series plot, the simulated and observational data exhibited very good agreement, especially in points such as maximum and minimum values, where the models performed very well in identifying these values (Fig 7). It is worth mentioning that the error values for all models followed a normal distribution. In the Hyper–arid–moderate, the MARS model had the lowest error (RMSE = 0.22) and the lowest standard deviation (St.D = 0.22). The error range was lower compared to the other two models in the respective figure. In the arid–cold and arid–hot climates, the LSSVR and MARS models had lower values (RMSE = 0.22, MSE = 0.052) and (RMSE = 0.27, MSE = 0.055) respectively (Fig 8). According to the figures, the MARS model had equal or sometimes better results than the other two models, which is attributed to its better performance in estimating the calibration data, which had more data points (Fig 9).

**Fig 10:**
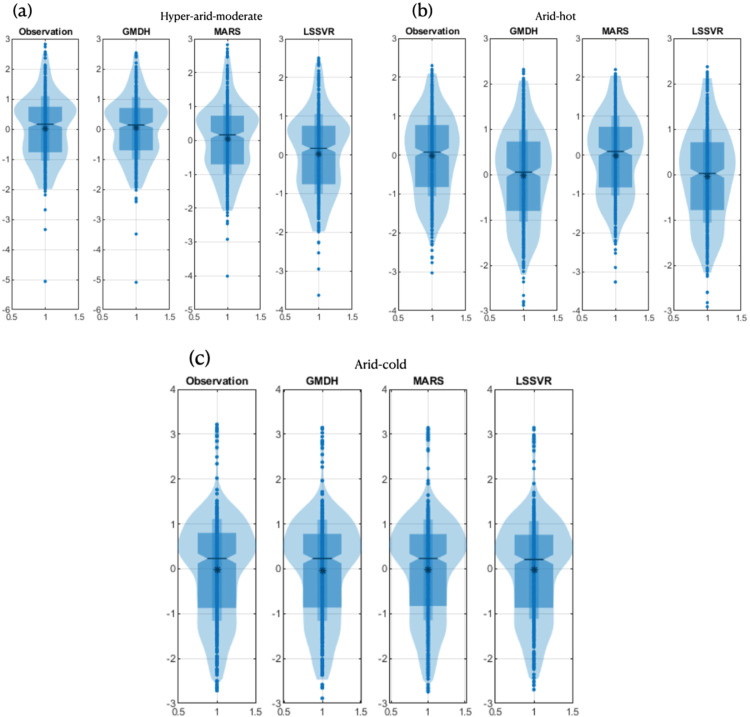
Boxplots for models used in different climates: a) hyper–arid–moderate, b) arid–cold, c) arid–hot climate.

In Fig 10, a visual summary of the data is presented in box plots, showing the data obtained from the models and observations. The figure illustrates that the space between the first and third quartiles of the data obtained from machine learning models is very close to the observational data. Additionally, the alignment of the median lines of these models with the observational data confirms the high accuracy of the machine learning models in estimating SPEI. An important point to note is that in the arid–cold climate, the MARS model had an outlier. However, in the arid–hot climate, both observational and simulated data had outliers, which the machine learning models are capable of identifying among the observational data. The max and min values in the hyper–arid–moderate and cold–arid climates were largely close to the values in the observational data, but in the arid–hot climate, the min values in both the MARS and GMDH models were higher than the observational data, while both max and min values in the LSSVR model are closer to the observational data.

**Fig 11:**
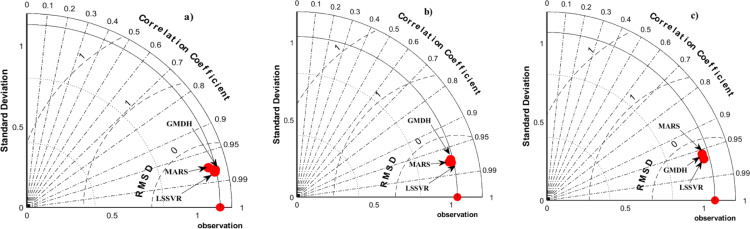
Taylor’s Diagram, a) hyper–arid–moderate, b) arid–cold, c) arid–hot climate.

For comparing the performance of the models, a Taylor diagram in Fig 11 was utilized. In this diagram, the performance of the models is evaluated using RMSE, Standard Deviation, Correlation Coefficient, and the proximity of model data to observational data. In the first stage, it was evident that the correlation coefficient between the machine–learning model data and observational data in all three climates ranged between 0.95 and 0.99, indicating a very good correlation (Fig 11a). Additionally, the RMSD for the data was also very low. In Figs 11b and 11c, the models have quite similar performance, but in Fig 11a, the MARS model is positioned slightly closer to the other two models. Based on this diagram, for the hyper–arid–moderate, arid–cold, and arid–hot regions, considering the discussed content and the position of the data relative to the observational data, the GMDH, LSSVR, and LSSVR models, respectively, had higher prediction accuracy. In general, the results of the Taylor diagram indicate that the machine learning models have a high prediction accuracy for the studied climates. Based on the results obtained, it is evident that all three models perform better in scenarios one and two. However, the results indicate that scenario one was the most effective for the arid–cold region among the three models used. This suggests that the performance of the signal parameters has enhanced forecast accuracy. Therefore, the utilization of large–scale signals can greatly aid in achieving more precise forecasting in certain regions.

**Fig 12:**
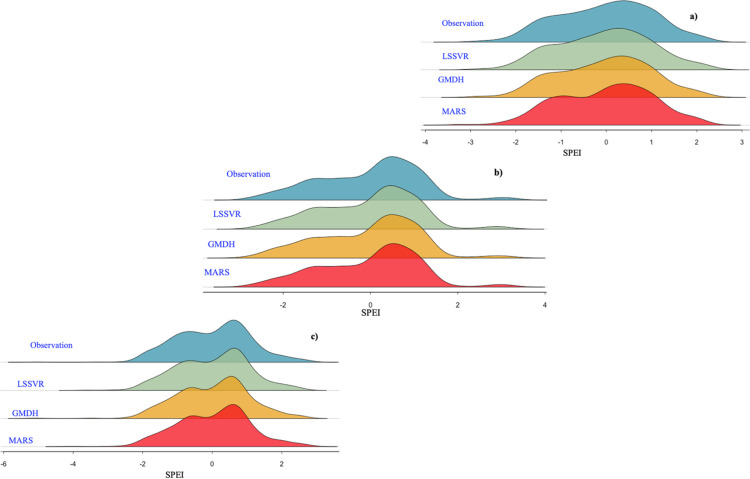
Regline’s diagram, a) arid–cold, b) hyper–arid–moderate, c) arid–hot climate.

In the Regline diagram (Fig 12a), the LSSVR model is much closer to the observed data in terms of appearance. Two other models have estimated SPEI values in the range (–1 and 0) lower than the observed values. In the hyper–arid–moderate climate (Fig 12b), the shape of the model data largely matches the observational data. In the arid–hot climate (Fig 12c), the models have estimated a large amount of data similar to the observation data, but the two LSSVR and MARS models have not been able to correctly estimate the minimum values, while the GMDH model has estimated these values with great accuracy.

**Fig 13:**
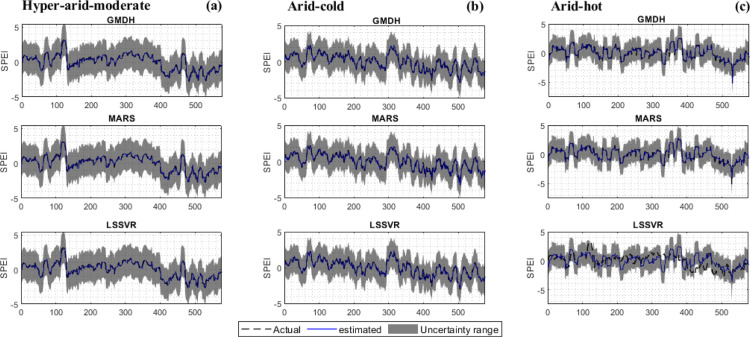
95% confidence interval of the best SPEI prediction scenarios for a) hyper–arid–moderate, b) arid–cold, c) arid–hot climate.

Fig 13 displays the predicted values along with the uncertainty band in all three climates using the selected models. Based on the graphs, the models have successfully estimated the SPEI values, aligning with the observed trend (Fig 13a–c). The predicted values in all climates fall within the acceptable uncertainty range. However, in the hot climate, values at step 530 slightly exceed the permissible uncertainty range, indicating increased uncertainty during severe droughts. While average SPEI values and predicted trends are within acceptable uncertainty levels, significant uncertainty remains in maximum and minimum values, warranting further investigation (Fig 13a–c). Overall, the models show minimal uncertainty in all climates, providing almost accurate estimates.

## 4. Discussion

The prediction results of the SPEI index indicated that the hyper–arid–moderate climate experienced a period of drought. In the arid and cold climate, the drought period began in 1998, and the most severe drought periods were experienced in 2006 and 2009. The arid and hot climate experienced less drought compared to the hyper–arid–moderate, and arid and cold climates. Therefore, the severity of drought varied in different climates and occurred at different times. This depends greatly on the changes in precipitation and temperature in the region. On the other hand, precipitation patterns and temperature changes also depend to a large extent on the topography of the region, sea surface temperature, and elevation of the region. This creates a complex system of various factors that affect drought. Hence, it is necessary for managers to devise specific strategies based on the type of climate. The results of this study can greatly assist managers in water and environmental resource management. By utilizing the findings of this research, water resources can be managed more effectively, and optimal allocation of surface and groundwater resources can be achieved. Appropriate management solutions and scenarios can be adopted, and with the help of water consumers, the needs of various regions can be managed under critical conditions.

In this study, three different models with different machine–learning structures were employed. The accuracy of their predictions showed that LSSVR, which has a relatively simpler structure, can be used. However, if a regression relationship model is desired, MARS is recommended. One of the special features of this model is its ability to extract regression relationships with accurate predictions. The results of this study demonstrated the successful performance of machine learning models for this problem, and MARS was preferred due to its better accuracy compared to other models, regardless of model structure. Therefore, this model is most suitable for a specific period and range, but results may vary slightly in different groups and ranges. Hence, investigating them in other ranges is deemed necessary. The consideration of uncertainty is among the important and necessary aspects of result analysis. Based on the results obtained, the models performed differently in each climate, serving as an example of the performance of GMDH. It can be concluded that the models’ performance varies according to different conditions. Their effectiveness is influenced by changes in input variables and the amount of SPEI, as well as the physical conditions of the area. While physics and regional conditions are typically not factored into machine learning models, regional conditions are indirectly considered through data changes. Consequently, no single model can be deemed superior without further investigation in this field. Understanding factors beyond parameter settings in machine learning models, such as changes in input parameters and regional conditions, can enhance the models’ performance in predicting SPEI. Considering the vegetation cover of areas could be an effective parameter for future research.

Uncertainty in the output of the results was assessed, and was found to be within an acceptable range. However, there is uncertainty in data collection, drought index calculation, and modeling knowledge, which can impact the results and SPEI values. Therefore, it is recommended that this issue be addressed in future research. The impact of climate change can be analyzed in predicting the duration and frequency of the aforementioned drought period, which is always recommended for further investigation in the continuation of this research. Climate change causes changes in precipitation patterns and temperature, which in turn have a significant impact on drought. This can lead to alterations in the frequency and duration of drought periods. Among the consequences of drought and climate change on water resources, one can mention the reduction of surface and groundwater resources, which has a significant impact on the water supply sources of the area. Additionally, the reduction of rainfall is one of the potential effects of droughts, which can challenge the recharge of the aquifer. Therefore, it is recommended to consider appropriate and drought–adapted management approaches. The limitations of this research include the unavailability of information in recent years (since 2015– 2024), as well as not considering the vegetation cover of the region due to limited access and uncertainty in input data. These limitations can be overcome by adopting appropriate approaches for future research and comparing the results with those of this study.

## 5. Conclusions

This study aimed to predict the SPEI–12 drought index in three climates: Hyper–arid–moderate, containing four meteorological stations; arid–cold, containing 6 stations; and arid–hot, containing 3 stations across Iran over a period from 1966 to 2014. For each climate, the best predictive model along with the most influential variables were identified. The predictive models included LSSVR, GMDH, and MARS. To predict the SPEI–12 index alongside meteorological data (Tmin, Tmax, Tmean, Pr, and ETPc), large–scale climate signal data (AO, NAO, PDO, and SOI) were used to assess their impact on predicting SPEI–12. The findings of this study showed that in Hyper–arid–moderate climate, the drought period began in 2000, as before that, the index was mostly above zero. In the arid and cold climate, the behavior was similar to the moderate and hyper–arid climate, and the most severe droughts occurred in 2006 and 2009. However, in the arid and hot climate, the SPEI–12 index had more oscillatory behavior, and a severe drought occurred in 2010. The use of large–scale climate signals alongside meteorological variables increased the accuracy of the models in predicting SPEI–12. Based on the extracted results in the arid–cold and arid–hot climates, the LSSVR had better forecasting performance with lower error evaluation criteria. For the Hyper–arid–moderate climates, GMDH shoved better forecasting accuracy than other methods. However, MARS also yielded acceptable forecasting results by providing regression relationships in all three climates. The findings of this study can contribute to better water management processes in regions like Iran and, consequently, facilitate sustainable water resource use by farmers and water stakeholders. By analyzing the results and forecasting the SPEI for future periods, it is possible to predict drought conditions. This prediction can then inform the implementation of appropriate strategies to meet water needs. Ultimately, this proactive approach can aid in effective water resource management. This research recommends exploring various perspectives, including the use of remote sensing combined with machine learning, gamma testing to select the best input combination, and examining the performance of MARS, LSSVR, and GMDH models in similar climates in different regions. These methods will help assess the comprehensiveness of the research findings.

## References

[1] Pereira LS, Cordery I, Iacovides I. Coping with water scarcity: Addressing the challenges. Springer Science & Business Media. 2009.

[2] Wilhite D, Sivakumar M, Pulwarty R. Managing drought risk in a changing climate: the role of national drought policy. Weather Clim Extrem. 2014;3:4–13.

[3] Burke EJ, Perry RH, Brown SJ. An extreme value analysis of UK drought and projections of change in the future. Journal of Hydrology. 2010;388(1–2):131–43.

[4] Djerbouai S, Souag–Gamane D. Drought forecasting using neural networks, wavelet neural networks, and stochastic models: case of the Algerois Basin in North Algeria. Water Resources Management. 2016;30:2445–64.

[5] Fung KF, Huang YF, Koo CH, Mirzaei M. Improved SVR machine learning models for agricultural drought prediction at downstream of Langat River Basin, Malaysia. Journal of Water and Climate Change. 2020;11(4):1383–98.

[6] Mendicino G, Senatore A, Versace P. Groundwater resource index (GRI) for drought monitoring and forecasting in a Mediterranean climate. Journal of Hydrology. 2008;357:282–302.

[7] Deo RC, Şahin M. Application of the artificial neural network model for prediction of monthly standardized precipitation and evapotranspiration index using hydrometeorological parameters and climate indices in eastern Australia. Atmospheric Research. 2015, 161, 65–81.

[8] Zhang Y, Yang H, Cui H, Chen Q. Comparison of the ability of ARIMA, WNN and SVM models for drought forecasting in the Sanjiang Plain, China. Natural Resources Research. 2020;29(2):1447–64.

[9] Esfahanian E, Nejadhashemi AP, Abouali M, Adhikari U, Zhang Z, Daneshvar F, et al. Development and evaluation of a comprehensive drought index. J Environ Manage. 2017;185:31–43. doi: 10.1016/j.jenvman.2016.10.050 28029478

[10] Begueria S, Vicente–Serrano S, Reig F, Latorre B. The standardized precipitation evapotranspiration index (SPEI) revisited: parameter fitting, evapotranspiration models, tools, datasets and drought monitoring. International Journal of Climatology. 2014;34(10):3001–23. doi: 10.1002/joc.3885

[11] Li X, He B, Quan X, Liao Z, Bai X. Use of the Standardized Precipitation Evapotranspiration Index (SPEI) to characterize the drying trend in Southwest China from 1982–2012. Remote Sensing. 2015;7(8):10917–37.

[12] Manatsa D, Mushore T, Lenouo A. Improved predictability of droughts over the Southern Africa using the Standardized precipitation evapotranspiration index and ENSO. Theoretical and Applied Climatology. 2015;127(1–2):259–74.

[13] Liu Z, Wang Y, Shao M, Jia X, Li X. Spatiotemporal analysis of multiscalar drought characteristics across the Loess Plateau of China. Journal of Hydrology. 2016;534:281–99.

[14] Maca P, Pech P. Forecasting SPEI and SPI Drought Indices Using the Integrated Artificial Neural Networks. Comput Intell Neurosci. 2016;2016:3868519. doi: 10.1155/2016/3868519 26880875 PMC4736223

[15] Paulo A, Pereira L. Prediction of SPI drought class transitions using Markov chains. Water Resources Management. 2009;21:1813–27.

[16] Abebe A, Foerch G. Stochastic simulation of the severity of hydrological drought. Water & Environment J. 2008;22(1):2–10. doi: 10.1111/j.1747-6593.2007.00080.x

[17] Alam ATMJ, Rahman MS, Sadaat AHM. Computational intelligence techniques in earth and environmental sciences. Springer, Dordrecht. 2014.

[18] Bazrafshan O, Salajegheh A, Bazrafshan J, Mahdavi M, Marj A. Hydrological drought forecasting using ARIMA models (case study: Karkheh Basin). Ecopersia. 2015;3:1099–117.

[19] Mossad A, Alazba A. Drought Forecasting Using Stochastic Models in a Hyper-Arid Climate. Atmosphere. 2015;6(4):410–30. doi: 10.3390/atmos6040410

[20] Ghordoyee Milan S, Kayhomayoon Z, Arya Azar N, Berndtsson R, Ramezani MR, Kardan Moghaddam H. Using machine learning to determine acceptable levels of groundwater consumption in Iran. Sustainable Production and Consumption. 2023;35:388–400. doi: 10.1016/j.spc.2022.11.018

[21] Kayhomayoon Z, Arya Azar N, Ghordoyee Milan S, Berndtsson R, Najafi Marghmaleki S. Application of soft computing and evolutionary algorithms to estimate hydropower potential in multi–purpose reservoirs. Applied Water Science. 2023;13(9):183. doi: insert_doi_here

[22] Arya Azar N, Ghordoyee Milan S, Kayhomayoon Z. The prediction of longitudinal dispersion coefficient in natural streams using LS-SVM and ANFIS optimized by Harris hawk optimization algorithm. J Contam Hydrol. 2021;240:103781. doi: 10.1016/j.jconhyd.2021.103781 33799017

[23] Arya Azar N, Kardan N, Ghordoyee Milan S. Developing the artificial neural network–evolutionary algorithms hybrid models (ANN–EA) to predict the daily evaporation from dam reservoirs. Engineering with Computers. 2023, 39(2), 1375–1393.

[24] Ali Z, Hussain I, Faisal M, Nazir HM, Hussain T, Shad MY, et al. Forecasting drought using multilayer perceptron artificial neural network model. Advances in Meteorology. 2017, 2017, 1–9

[25] Kousari MR, Hosseini ME, Ahani H, Hakimelahi H. Introducing an operational method to forecast long–term regional drought based on the application of artificial intelligence capabilities. Theoretical and Applied Climatology. 2017;127:361–80.

[26] Seibert M, Merz B, Apel H. Seasonal forecasting of hydrological drought in the Limpopo Basin: a comparison of statistical methods. Hydrology and Earth System Sciences. 2017;21:1611–29. doi: 10.5194/hess-21-1611-2017

[27] Fung KF, Huang YF, Koo CH. Coupling fuzzy–SVR and boosting–SVR models with wavelet decomposition for meteorological drought prediction. Environmental Earth Sciences. 2019;78(1):1–18.

[28] Khan N, Sachindra DA, Shahid S, Ahmed K, Shiru MS, Nawaz N. Prediction of droughts over Pakistan using machine learning algorithms. Advances in Water Resources. 2020;139 103562.

[29] Shamshirband S, Hashemi S, Salimi H, Samadianfard S, Asadi E, Shadkani S, et al. Predicting standardized streamflow index for hydrological drought using machine learning models. Engineering Applications of Computational Fluid Mechanics. 2020;14(1):339–50.

[30] Deo RC, Kisi O, Singh VP. Drought forecasting in eastern Australia using multivariate adaptive regression spline, least square support vector machine and M5Tree model. Atmospheric Research. 2017;184:149–75.

[31] Cordery I, McCall M. A model for forecasting drought from teleconnections. Water Resources Research. 2000;36(3):763–76.

[32] Deo RC, Salcedo–Sanz S, Carro–Calvo L, Saavedra–Moreno B. Drought prediction with standardized precipitation and evapotranspiration index and support vector regression models. Integrating disaster science and management. 2018:151–74.

[33] Tian Y, Xu Y-P, Wang G. Agricultural drought prediction using climate indices based on Support Vector Regression in Xiangjiang River basin. Sci Total Environ. 2018;622–623:710–20. doi: 10.1016/j.scitotenv.2017.12.025 29223897

[34] Morid S, Smakhtin V, Bagherzadeh K. Drought forecasting using artificial neural networks and time series of drought indices. International Journal of Climatology: A Journal of the Royal Meteorological Society. 2007;27(15):2103–11.

[35] Canon J, Gonzalez J, Valde’s J. Precipitation in the Colorado River Basin an Its Low Frequency Associations With PDO and ENSO Signals. Journal of Hydrology. 2007;344:252–64.

[36] Fernandes VJ, de Louw PG, Bartholomeus RP, Ritsema CJ. Machine learning for faster estimates of groundwater response to artificial aquifer recharge. Journal of Hydrology. 2024;637 131418.

[37] Singh AK, Kumar P, Ali R, Al–Ansari N, Vishwakarma DK, Kushwaha KS, et al. An integrated statistical–machine learning approach for runoff prediction. Sustainability. 2022;14(13):8209.

[38] Trach R, Trach Y, Kiersnowska A, Markiewicz A, Lendo–Siwicka M, Rusakov K. A study of assessment and prediction of water quality index using fuzzy logic and ANN models. Sustainability. 2022;14(9):5656.

[39] Najafzadeh M, Oliveto G, Saberi-Movahed F. Estimation of scour propagation rates around pipelines while considering simultaneous effects of waves and currents conditions. Water. 2022;14(10):1589.

[40] Kayhomayoon Z, Jamnani MR, Rashidi S, Milan SG, Azar NA, Berndtsson R. Soft computing assessment of current and future groundwater resources under CMIP6 scenarios in northwestern Iran. Agricultural Water Management. 2023;285:108369.

[41] Kumar V, Kedam N, Sharma KV, Mehta DJ, Caloiero T. Advanced machine learning techniques to improve hydrological prediction: A comparative analysis of streamflow prediction models. Water. 2023;15(14):2572. doi: insert_doi_here

[42] Poornima S, Pushpalatha M. Drought prediction based on SPI and SPEI with varying timescales using LSTM recurrent neural network. Soft Computing. 2019;23:8399–412.

[43] Dikshit A, Pradhan B, Huete A. An improved SPEI drought forecasting approach using the long short-term memory neural network. J Environ Manage. 2021;283:111979. doi: 10.1016/j.jenvman.2021.111979 33482453

[44] Mokhtar A, Jalali M, He H, Al-Ansari N, Elbeltagi A, Alsafadi K, et al. Estimation of SPEI Meteorological Drought Using Machine Learning Algorithms. IEEE Access. 2021;9:65503–23. doi: 10.1109/access.2021.3074305

[45] Shang J, Zhao B, Hua H, Wei J, Qin G, Chen G. Application of informer model based on SPEI for drought forecasting. Atmosphere. 2023, 14(6), 951.

[46] Soh Y, Koo C, Huang Y, Fung K. Application of artificial intelligence models for the prediction of standardized precipitation evapotranspiration index (SPEI) at Langat River Basin, Malaysia. Computers and Electronics in Agriculture. 2018;144:164–73.

[47] Abbasi A, Khalili K, Behmanesh J, Shirzad A. Drought monitoring and prediction using SPEI index and gene expression programming model in the west of Urmia Lake. Theoretical and Applied Climatology. 2019;138:553–67.

[48] Xu D, Zhang Q, Ding Y, Zhang D. Application of a hybrid ARIMA-LSTM model based on the SPEI for drought forecasting. Environ Sci Pollut Res Int. 2022;29(3):4128–44. doi: 10.1007/s11356-021-15325-z 34403057

[49] Ghasemi P, Karbasi M, Nouri AZ, Tabrizi MS, Azamathulla HM. Application of Gaussian process regression to forecast multi–step ahead SPEI drought index. Alexandria Engineering Journal. 2021;60(6):5375–92.

[50] Tian W, Wu J, Cui H, Hu T. Drought prediction based on feature–based transfer learning and time series imaging. IEEE Access. 2021;9:101454–68.

[51] Karbasi M, Karbasi M, Jamei M, Malik A, Azamathulla HM. Development of a new wavelet–based hybrid model to forecast multi–scalar SPEI drought index (case study: Zanjan city, Iran). Theoretical and Applied Climatology. 2022;147:499–522.

[52] Lotfirad M, Esmaeili–Gisavandani H, Adib A. Drought monitoring and prediction using SPI, SPEI, and random forest model in various climates of Iran. Journal of Water and Climate Change. 2022;13(2):383–406.

[53] Danandeh Mehr A, Rikhtehgar Ghiasi A, Yaseen ZM, Sorman AU, Abualigah L. A novel intelligent deep learning predictive model for meteorological drought forecasting. Journal of Ambient Intelligence and Humanized Computing. 2023;14(8):10441–55. doi: 10.1007/s12652-023-04234-5

[54] Khalili A, Rahimi J. High–resolution spatiotemporal distribution of precipitation in Iran: a comparative study with three global–precipitation datasets. Theoretical and Applied Climatology. 2014;118:211–21.

[55] Liu W, Zhu S, Huang Y, Wan Y, Wu B, Liu L. Spatiotemporal variations of drought and their teleconnections with large–scale climate indices over the Poyang Lake Basin, China. Sustainability. 2020;12(9):3526. doi: 10.3390/su12093526

[56] Kao H, Yu J. Contrasting Eastern–Pacific and Central–Pacific types of El Niño. Journal of Climate. 2009;22:615–32.

[57] Kushnir Y, Robinson WA, Bladé I, Hall NMJ, Peng S, Sutton R. Atmospheric GCM response to extratropical SST anomalies: Synthesis and evaluation. Journal of Climate. 2002;15(16):2233–56.

[58] Vicente–Serrano SM, Beguería S, López–Moreno JI. A multiscalar drought index sensitive to global warming: the standardized precipitation evapotranspiration index. Journal of Climate. 2010;23(7):1696–718. doi: 10.1175/2010JCLI3672.1

[59] Friedman JH. Multivariate adaptive regression splines. The Annals of Statistics. 1991;19(1):1–67. doi: 10.1214/aos/1176347963

[60] Najafzadeh M, Anvari S. Long-lead streamflow forecasting using computational intelligence methods while considering uncertainty issue. Environ Sci Pollut Res Int. 2023;30(35):84474–90. doi: 10.1007/s11356-023-28236-y 37369900

[61] Ivakhnenko AG. The group method of data handling; a rival of the method of stochastic approximation. Soviet Automatic Control c/c of Avtomatika. 1968;1:43–55.

[62] Moshizi ZGN, Bazrafshan O, Etedali HR, Esmaeilpour Y, Collins B. Application of inclusive multiple model for the prediction of saffron water footprint. Agricultural Water Management. 2023;277(1):108125. doi: 10.1016/j.agwat.2023.108125

